# An International Terminology for Endometriosis, 2021 †,‡

**DOI:** 10.52054/FVVO.13.4.036

**Published:** 2021-12-30

**Authors:** International Working Group of AAGL, ESGE, ESHRE and WES, C Tomassetti, N.P. Johnson, J Petrozza, M.S. Abrao, J.I. Einarsson, A.W. Horne, T.T.M. Lee, S Missmer, N Vermeulen, K.T. ZonderVan, G Grimbizis, R.L. de Wilde

**Affiliations:** University Hospital Leuven, Department of Obstetrics and Gynaecology, Leuven University Fertility Centre, Leuven, Belgium; Robinson Research Institute, University of Adelaide, Adelaide, South Australia; Massachusetts General Hospital Fertility Center, Department of Obstetrics and Gynecology, Boston, MA, USA; Disciplina de Ginecologia, Departamento de Obstetricia e Ginecologia, Faculdade de Medicina FMUSP, Universidade de Sao Paulo, São Paulo, SP, Brazil; Gynecologic Division, BP - A Beneficencia Portuguesa de Sao Paulo, São Paulo, SP, Brazil; Brigham and Women’s Hospital, Department of Obstetrics and Gynecology, Division of Minimally Invasive Gynecologic Surgery, Boston, MA, USA; University of Edinburgh, MRC Centre for Reproductive Health, QMRI, 49 Little France Crescent, Edinburgh, UK EH16 4TJ; Magee Womens Hospital of UPMC, Department of Obstetrics, Gynecology and Reproductive Sciences, Pittsburgh, PA, USA; Michigan State University College of Human Medicine, Department of Obstetrics, Gynecology and Reproductive Biology, East Lansing, MI, USA; Harvard University T H Chan School of Public Health, Department of Epidemiology, Boston, MA, USA; World Endometriosis Research Foundation, WERF, London, UK; ESHRE, Central office, Meerstraat 60, Grimbergen, BE 1852, Belgium; University of Oxford, Oxford Endometriosis CaRe Centre, Nuffield Department of Women’s & Reproductive Health, Oxford, Oxfordshire, UK; University of Oxford, Wellcome Centre for Human Genetics, Oxford, Oxfordshire, UK; Medical School, Aristotle University of Thessaloniki, 1st Dept Obstet Gynecol, Tsimiski 51 Street, Thessaloniki, Greece 54623; Carl von Ossietzky Universitat Oldenburg, University Hospital for Gynecology, Oldenburg, Germany

**Keywords:** Endometriosis, glossary, terminology, endometrioma, excision, ablation, coagulation

## Abstract

**Background:**

Different classification systems have been developed for endometriosis, using different definitions for the disease, the different subtypes, symptoms and treatments. In addition, an International Glossary on Infertility and Fertility Care was published in 2017 by the International Committee for Monitoring Assisted Reproductive Technologies (ICMART) in collaboration with other organisations. An international working group convened over the development of a classification or descriptive system for endometriosis. As a basis for such system, a terminology for endometriosis was considered a condition sine qua non.

**Objectives:**

The aim of the current paper is to develop a set of terms and definitions on endometriosis that would be the basis for standardisation in disease description, classification and research.

**Materials and Methods:**

The working group listed a number of terms relevant to be included in the terminology, documented currently used and published definitions, and discussed and adapted them until consensus was reached within the working group. Following stakeholder review, further terms were added, and definitions further clarified. Although definitions were collected through published literature, the final set of terms and definitions is to be considered consensus-based. After finalisation of the first draft, the members of the international societies and other stakeholders were consulted for feedback and comments, which led to further adaptations.

**Results:**

A list of 49 terms and definitions in the field of endometriosis is presented, including a definition for endometriosis and its subtypes, different locations, interventions, symptoms and outcomes. Endometriosis is defined as a disease characterised by the presence of endometrium-like epithelium and/or stroma outside the endometrium and myometrium, usually with an associated inflammatory process.

**Conclusions:**

The current paper outlines a list of 49 terms and definitions in the field of endometriosis. The application of the defined terms aims to facilitate harmonisation in endometriosis research and clinical practice. Future research may require further refinement of the presented definitions.

**What is new?:**

A consensus based international terminology for endometriosis for clinical and research use.

## Introduction

Endometriosis is considered a spectrum disease with a variety of subtypes and clinical presentations. One consequence of this ambiguity is a large heterogeneity in published studies that are either evaluating diagnostic and therapeutic interventions in endometriosis patients in general, or investigating a certain subgroup based on a published classification, or a disease subtype, with a study-specific definition. The resulting heterogeneity makes it difficult to interpret and summarise published data and draw conclusions on best practice in care for patients with endometriosis.

The need for standardisation has been repeatedly mentioned by experts in the field (Meuleman et al., 2011; Rogers et al., 2013). The lack of a uniform and widely accepted terminology for endometriosis has created difficulties in standardising and in comparing interventions and outcomes. This has further led to difficulty in defining clinical recommendations for endometriosis management (Nisolle and Donnez, 1997).

In absence of a specific terminology of endometriosis, definitions for endometriosis have been included in international glossaries, such as the International Glossary on Infertility and Fertility Care (Zegers-Hochschild et al., 2017), the International Statistical Classification of Diseases and Related Health Problems (ICD) published by the World Health Organization (WHO) [https://www.who.int/classifications/icd/icdonlineversions/en/], and other recent publications attempting standardisation (Vanhie et al., 2016; Johnson et al., 2017). However, the definitions are either not very detailed or elaborate, not widely accepted or not comprehensive for endometriosis. This paper describes a terminology for endometriosis prepared by an international working group representing four international societies with a focus on endometriosis [American Association of Gynaecologic Laparoscopists (AAGL), European Society for Gynecological Endoscopy (ESGE), European Society of Human Reproduction and Embryology (ESHRE) and World Endometriosis Society (WES)].

## Materials and Methods

The current paper is a consensus paper, predominantly based on the opinion of the working group members. The working group constructed a list of terms to be defined on different topics, including endometriosis and its subtypes, locations of the endometriosis lesions, treatments and interventions, and outcome parameters. Published literature and information was collected for the different terms, and definitions were extracted from the key papers. All collected definitions were discussed and, where needed, adapted to fit the aim of the current paper. Whenever definitions were significantly adapted, a justification for the adaptations was formulated in the results section. Before finalisation of the paper, a stakeholder review was organised. The collaborating organisations and individual experts formulated a total of 160 comments, which were tabulated and discussed by the working group. Where relevant, corrections, clarifications and adaptations were made to the text and the terms listed. The review report is available on the societies’ websites.

## Results

As a starting point for standardisation and to be able to universally use the classification, definitions of terms were structured in four sections: endometriosis, subtypes and locations (Table I); anatomical spaces and other locations where endometriosis can be detected (Table II); endometriosis treatments and interventions (Table III); and outcome parameters (Table IV). For symptoms associated with endometriosis, the definitions are generally clear and can be consulted in other papers (Zegers-Hochschild et al., 2017; Vitonis et al., 2014).

**Table I:** Terms and definitions for endometriosis, subtypes and locations.

Term	Definition	Source
Endometriosis	A disease characterised by the presence of endometrium-like epithelium and/or stroma outside the endometrium and myometrium, usually with an associated inflammatory process.	Adapted from (Whitaker, et al., 2019) (Johnson et al., 2017) (Zegers-Hochschild et al., 2017)
Peritoneal / superficial endometriosis	Endometrium-like tissue lesions involving the peritoneal surface. The lesions can have different appearances and colour e.g. clear, black, etc.	Adapted from (International Classification of Diseases and Related Health Problems (ICD-11), 2020)
Ovarian endometriotic cyst / endometrioma	Endometrium-like tissue in the form of ovarian cysts. They may be either invagination cysts or true cysts with the cyst wall also containing endometrium-like tissue and dark blood-stained fluid, the colour and consistency of which gives rise to the name ‘chocolate cysts’.	Adapted from (Whitaker et al., 2019)
Deep endometriosis	Endometrium-like tissue lesions in the abdomen, extending on or under the peritoneal surface. They are usually nodular, able to invade adjacent structures, and associated with fibrosis and disruption of normal anatomy.	Adapted from (Cornillie et al., 1990, Johnson et al., 2017, Koninckx and Martin, 1992, Whitaker et al., 2019, Zegers-Hochschild et al., 2017)
Bowel endometriosis	Endometriosis situated inside the bowel wall. Although mostly affecting the rectosigmoid area, lesions can be found also in other parts of the gastrointestinal system, including the appendix. Lesions on the peritoneal surface of the bowel are considered peritoneal endometriosis.	Adapted from (International Classification of Diseases and Related Health Problems (ICD-11))
Bladder endometriosis	Endometriosis involving the detrusor muscle and/or the bladder epithelium. Lesions on the peritoneal surface of the bladder are considered peritoneal endometriosis.	
Extra-abdominal endometriosis	Endometrium-like tissue outside the abdominal cavity.	
Iatrogenic endometriosis	Lesions resulting from direct or indirect dissemination of endometrium during surgery.	
Adhesions (peritoneal)	Bands of fibrous scar tissue that may bind the abdominal and pelvic organs, including the intestines and peritoneum, to each other. They can be dense and thick or filmy and thin. Adhesions can be induced by endometriosis as a result of the inflammatory process of the disease.	Adapted from (Zegers-Hochschild et al., 2017)

**Table II:** Terms and definitions for anatomical spaces and other locations where endometriosis can be detected.

Term	Definition	Source
Pararectal space	The retroperitoneal space lying lateral to the rectum on either side. The ureter further divides the pararectal space into the medial pararectal space (Okabayashi space) and lateral pararectal space (Latzko space).	(Puntambekar and Manchanda, 2018)
Paravesical space	The retroperitoneal space that lies laterally to the urinary bladder and anterior and superior to the pararectal space.	(Puntambekar and Manchanda, 2018)
Pouch of Douglas (or Recto-uterine pouch) – Cul-de-Sac	The space between the posterior uterus and the anterior rectum. It is bordered laterally by the rectouterine folds, peritoneal folds that extend from the rectum to the posterior broad ligament at the cervix.	(Heller, 2016)
Presacral space	A thin, small retroperitoneal space lying behind the rectum is covered by the mesorectum anteriorly and Waldeyer fascia posteriorly.	(Puntambekar and Manchanda, 2018)
Prevesical space	A small midline retroperitoneal space that lies between the bladder and the anterior abdominal wall. It communicates with the paravesical space on both sides and is enclosed laterally by the lateral umbilical ligament, which is the continuation of the obliterated hypogastric artery onto the abdominal wall	(Puntambekar and Manchanda, 2018)
Rectovaginal space	The area behind the pouch of Douglas, enclosed anteriorly by the uterus and the posterior vaginal wall, posteriorly by the rectum, and laterally by the uterosacral and the Mackenrodt ligament	(Puntambekar and Manchanda, 2018)
Retrocervical area	The area behind the cervix and above the rectovaginal septum.	
Retropubic Space or Space of Retzius	The anatomic space containing areolar connective tissue between the back of the pubic bone and the anterolateral portion of the bladder.	Adapted from (Rogers Jr, 2007)
Uterosacral ligaments	The ligaments from the posterior aspect of the uterus to the sacrum.	
Vesicovaginal space	The space found between the anterior surface of the vagina and the posterior aspect of the bladder down to the trigone. The space is bordered laterally by the bladder “pillars” that allow for the passage of the inferior vesical arteries, veins and ureter to the bladder.	(Rogers Jr, 2007)
Rectum	The concluding part of the large intestine that terminates in the anus and measures 12–15 cm in length.	(Beck et al., 2011)

**Table III:** Terms and definitions for treatments and interventions used in the context of endometriosis.

Term	Definition	Source
Reproductive surgery	Surgical procedures performed to diagnose, conserve, correct and/or improve reproductive function. Surgery for contraceptive purposes, such as tubal ligation, are also included within this term.	Adapted from (Zegers- Hochschild et al., 2017)
Superficial excision	Superficial excision of serosal and subserosal endometriosis (mechanically, with electrosurgery, laser or other energy source) that does not require suturing/closure.	(Vanhie et al., 2016)
Partial thickness discoid excision	Selective excision of the bowel/bladder endometriosis lesion (mechanically, with electrosurgery, laser or other energy source) without entering the bowel/bladder lumen, that requires suturing/closure (i.e. closure of a muscularis defect without a mucosal defect in the bowel wall). Shaving is a form of partial thickness discoid excision.	Adapted from (Vanhie et al., 2016)
Full thickness discoid excision	Selective excision of the bowel endometriosis lesion (mechanically, with electrosurgery, laser or other energy source) with opening of the bowel lumen followed by closure of the bowel. Subtypes: (1) Open full thickness disc excision: excision with opening of lumen followed by closure (2) Closed full thickness disc excision: excision with stapler	(Vanhie et al., 2016)
Bowel resection and re-anastomosis	Resection of a bowel segment affected by endometriosis followed by re-anastomosis by any means.	(Vanhie et al., 2016)
Bladder wall resection	Selective excision of the bladder endometriosis lesion (mechanically, with electrosurgery, laser or other energy source) with or without opening of the bladder lumen. Subtypes: (1) Partial thickness bladder resection without opening of the bladder lumen requiring suturing. (2) Full thickness bladder wall resection (partial cystectomy) with opening of the bladder lumen requiring suturing and closure of the bladder wall.	Adapted from (Vanhie et al., 2016)
Cystectomy (total)	Excision of the cyst wall mechanically by gentle traction and counter-traction to dissect the capsule from the ovarian parenchyma. Electrosurgery, laser, haemostatic agents, and/or other energy sources could be used to facilitate the process and to provide haemostasis.	Adapted from (Working group of ESGE, ESHRE and WES, 2017a, Working group of ESGE, ESHRE and WES, 2017b)
Partial ovarian cystectomy	A combination of excisional and ablative surgery. A large part of the endometrioma is first excised according to the cystectomy technique, followed by vaporisation of the remaining endometrioma close to the hilus using energy such as electrosurgery	Adapted from (Donnez et al., 2010)
Ablation	Obliteration of the inner surface of the cyst wall in cases of endometriomas and/or endometriotic lesions in cases of peritoneal endometriosis using, electro- or ultrasound high frequency-modes, laser, or plasma energy	Adapted from (Working group of ESGE, ESHRE and WES, 2017a, Working group of ESGE, ESHRE and WES, 2017b)
Coagulation or Fulguration or	Destruction of the inner surface of the cyst wall in cases of endometriomas and/or endometriotic lesions in cases of peritoneal endometriosis using electrosurgery.	
Ureterolysis	Selective dissection of the ureter from a lesion, either mechanically or with electrosurgery, laser or any other energy source. Restoration of the anatomy of the ureter intending to restore normal function through lysis and/or resection of adhesions. Subtypes: (1) Without opening of the ureteric wall (2) With opening and re-suturing of the ureteric wall.	Adapted from (Vanhie et al., 2016)
Ureteral segmental resection	Resection of a ureteral segment affected by endometriosis followed by ipsilateral uretero-ureteral re-anastomosis or ureteral reimplantation into the bladder.	(Vanhie et al., 2016)
Medically assisted reproduction (MAR)	Reproduction brought about through various interventions, procedures, surgeries and technologies to treat different forms of fertility impairment and infertility. These include ovulation induction, ovarian stimulation, ovulation triggering, all assisted reproductive technology (ART) procedures, uterine transplantation and intra-uterine, intracervical and intravaginal insemination with semen of husband/partner or donor.	(Zegers-Hochschild et al., 2017)
Fertility preservation	Various interventions, procedures and technologies, including cryopreservation of gametes, embryos or ovarian tissue, to preserve reproductive capacity.	Adapted from (Zegers-Hochschild et al., 2017)

**Table IV:** Terms and definitions for treatments and interventions used in the context of endometriosis.

Term	Definition	Source
Core outcomes in endometriosis	A set of thirteen core outcomes identified for endometriosis trials. - Core outcomes for pain and quality of life (3) are: overall pain, improvement in the most troublesome symptom, and quality of life. - Core outcomes for infertility (8) include: viable intrauterine pregnancy confirmed by ultrasound, pregnancy loss, termination of pregnancy, live birth, time to pregnancy leading to live birth, gestational age at delivery, birthweight, neonatal mortality, and major congenital abnormalities. - Two core outcomes applicable to all endometriosis trials are: adverse events and patient satisfaction with treatment.	(Vanhie et al., 2016)
Fertility	The capacity to establish a clinical pregnancy.	(Zegers-Hochschild et al., 2017)
Infertility	A disease characterized by the failure to establish a clinical pregnancy after 12 months of regular, unprotected sexual intercourse or due to an impairment of a person’s capacity to reproduce either as an individual or with his/her partner.	Adapted from (World Health Organization, 2018, Zegers-Hochschild et al., 2017)
Endometriosis-associated infertility	Impaired fertility in which the female has a prior diagnosis of endometriosis.	
Pregnancy	A state of reproduction beginning with implantation of an embryo in a woman and ending with the complete expulsion and/or extraction of all products of implantation.	(Zegers-Hochschild et al., 2017)
Pain	Various different pain patterns have been described for endometriosis including dysmenorrhoea (menstrual period pain), dyspareunia (pain related to sexual activity), dyschezia (bowel related pain), dysuria (urinary tract related pain), mid-cycle pain (mittelschmerz) that is often related to ovulation, non-cyclic pelvic pain.	Adapted from (Vanhie et al., 2016)
Quality of life (QoL)	The “individuals’ perceptions of their position in life in the context of the culture and value systems in which they live and in relation to their goals, expectations, standards and concerns”. It is a broad ranging concept incorporating in a complex way the persons’ physical health, psychological state, level of independence, social relationships, personal beliefs and their relationships to salient features of the environment. Numerous quality of life measures are available.	(World Health Organization, 1998)
Complication (GRADE I)	Any deviation from the normal postoperative course without the need for pharmacological treatment or surgical, endoscopic or radiological interventions. Allowed therapeutic regimens are: drugs as antiemetics, antipyretics, analgesics, diuretics, electrolytes, and physiotherapy. This grade also includes wound infections opened at the bedside.	(Dindo et al., 2004)
Complication (GRADE II)	Requiring pharmacological treatment with drugs other than such allowed for grade I complications. Blood transfusions and total parenteral nutrition are also included.	(Dindo et al., 2004)
Complication (GRADE III)	Requiring surgical, endoscopic or radiological intervention. Grade IIIa: Intervention not under general anaesthesia. Grade IIIb: Intervention under general anaesthesia.	(Dindo et al., 2004)
Complication (GRADE IV)	Life-threatening complication (including central nervous system complications)* requiring IC/ICU management. Grade IVa: Single organ dysfunction (including dialysis). Grade IVb: Multiorgan dysfunction.	(Dindo et al., 2004)
Complication (GRADE V)	Death of a patient	(Dindo et al., 2004)
Sequelae	An ‘after-effect’ of surgery that is inherent to the procedure, e.g. inability to conceive after removing the uterus	(Vanhie et al., 2016)
Recurrence	Lesion recurrence on reoperation or imaging after previous complete excision of the disease. (1) Symptom based suspected recurrence: Symptom recurrence based on patient history, but not proven/confirmed by imaging and/or surgery (2) Imaging based suspected recurrence: Endometriosis recurrence based on imaging (in patients with or without symptoms). (3) Laparoscopically proven recurrence: Recurrence of visual endometriosis without histological proof: during laparoscopy endometriosis is visually observed but either not biopsied or biopsied without histologically proven endometriosis. (4) Histologically proven recurrence: Recurrence of histologically proven endometriosis: during laparoscopy endometriosis is visually observed and confirmed histologically.	Adapted from (Vanhie et al., 2016)
Residual disease	Endometriosis lesions not completely removed at the time of surgery.	

*Brain haemorrhage, ischemic stroke, subarachnoid bleeding, but excluding transient ischemic attacks.

The terms and definitions are listed in Tables I-IV.

### Endometriosis and its subtypes

For endometriosis, previous definitions have focussed on pathology or on the symptoms suffered by those with the disease. The WES definition, a strong consensus from 55 expert representatives of 29 national and international organisations (which was considered unanimous, where fewer than 5% of experts disagreed with the definition) introduced the concept that symptoms are an important aspect of ‘disease’ suffered by patients. Without symptoms, the occurrence of lesions per se might not necessarily be considered a disease, given that occurrence of lesions in the absence of symptoms may be considered a ubiquitous finding (Johnson et al., 2017; Koninckx et al., 1994). A more comprehensive, contemporary characterisation of endometriosis has been provided through WES consensus that alludes to other essential elements including incidence, pathogenesis, multifactorial aetiology including genetic factors with possible epigenetic influences, possible effects of environmental exposures, pain syndrome elements, proliferative nature, hormone responsiveness (oestrogen-dependence and progesterone-resistance), and overlap with other conditions characterised by pelvic–abdominal pain and infertility (https://endometriosis.ca/endometriosis/). In the International Glossary on Infertility and Fertility Care, endometriosis is defined as a disease characterised by the presence of endometrium-like epithelium and stroma outside the endometrium and myometrium, with further specification that intrapelvic endometriosis can be located superficially on the peritoneum (peritoneal endometriosis), can extend 5mm or more beneath the peritoneum (deep endometriosis) or can be present as an ovarian endometriotic cyst (endometrioma) (Zegers-Hochschild et al., 2017). For the current terminology, it was decided to focus on the pathology, and define endometriosis- associated symptoms separately. The definition from the International Glossary on Infertility and Fertility Care was further adapted with addition of the most important characteristic of endometriosis “inflammatory”, in line with a recent WHO document stating that endometriosis causes a chronic inflammatory reaction that may result in the formation of scar tissue (adhesions, fibrosis) within the pelvis and other parts of the body (WHO, 2021). The specificities of the subtypes were removed from the definition, as they are defined separately (Table I). Recent observations suggest to focus on the fibrotic nature of the disease in its definition (Vigano et al., 2018), but further evidence is needed before such adaptation can be made.

For peritoneal or superficial endometriosis, some cases may only be identified following microscopic histological assessment of macroscopically normal peritoneum. This concept includes that the presence of endometrial-like tissue, and even if only endometrial stroma is found by the pathologist, can be considered endometriosis (Abrao et al., 2003). However, this was not considered a relevant addition to the definition. A consensus was reached to adapt the definition from the International Classification of Diseases and Related Health Problems (ICD) (ICD-11, 2020), reading “superficial endometriosis of pelvic peritoneum is characterised by ectopic growth and function of endometrial tissue extending 5 millimetres or less under the visceral or parietal pelvic peritoneal surface and appearing as black- brown or light red-orange lesions.” Existing definitions, such as this one, typically define the depth of the lesions and provide examples of the appearances of them. With regard to the depth, it was argued that the depth of the lesions cannot be accurately measured (in mm). Alternatively, it can be assessed whether the lesion is on the peritoneal surface or under the surface, and this could be integrated in the definition. With regards to the appearances of the lesions, it was considered that a specific list of appearances would never be exhaustive and a general statement was included.

Deep endometriosis is historically defined as extending 5mm under the peritoneal surface (Zegers-Hochschild et al., 2017; Johnson et al., 2017; Whitaker et al., 2019; Koninckx and Martin, 1992). As argued for peritoneal disease, assessing the depth of the infiltration cannot accurately be measured, and therefore it was decided to remove this from the definition.

The definition of Whitaker and colleagues (including elements from the ICD code) for ovarian endometriosis (cystic) or endometrioma was slightly rephrased, similar to the definition of peritoneal and deep endometriosis (Whitaker et al., 2019). As it is not clear whether endometrioma are invagination cysts or true cysts, it was decided to keep both in the definition.

Although not subtypes, a definition was added for bowel and bladder endometriosis. Fallopian tube, pelvic sidewall and other lesions are to be included as peritoneal endometriosis or deep endometriosis, depending on the depth of the lesions.

For extrapelvic endometriosis, there was consensus in the group that it should be defined as a separate entity. With regards to defining the possible locations, it was considered that vaginal disease and diaphragmatic disease may be extended abdominal endometriosis (in analogy with oncological definitions). For all other locations (outside the abdominal cavity), it was agreed to use the term extra-abdominal endometriosis and to define it as endometrium-like tissue outside the abdominal cavity.

Similarly, there was consensus to define iatrogenic endometriosis as an endometriosis subtype. The definition was formulated as lesions resulting from direct or indirect dissemination of endometrium following during surgery. Iatrogenic endometriosis has various manifestations resulting from different surgical procedures. The most common form of iatrogenic endometriosis is abdominal wall endometriosis - commonly involving the skin or subcutaneous layer of abdominal wall, but it can also involve the fascia and muscular layer - following Caesarean section. Other manifestations include episiotomy scar endometriosis or laparoscopic trocar site endometriosis, which involves various layers of abdominal wall, and endometriosis implants at various locations in the abdomen including peritoneum and visceral structures, such as bowel or bladder, attributed to mechanical uterine morcellation.

Finally, (peritoneal) adhesions were defined based on the definition from the International Glossary on Infertility and Fertility Care, i.e. bands of fibrous scar tissue that may bind the abdominal and pelvic organs, including the intestines and peritoneum, to each other. They can be dense and thick or filmy and thin (Zegers-Hochschild et al., 2017). Within the context of endometriosis, adhesions can result from the inflammatory process of the disease and this was specified in the definition.

### Adenomyosis

Adenomyosis is defined by the International Glossary on Infertility and Fertility Care as a form of endometriosis marked by the presence of endometrium-like epithelium and stroma outside the endometrium in the myometrium (Zegers- Hochschild et al., 2017). Different theories have been postulated with regards to adenomyosis and whether or not it is a subtype of endometriosis or a different entity. The first theory was based on similar features between endometriosis and adenomyosis and the fact that they often coexist in the same patient. However, recent reports suggest the theory of two different entities because of specific pathogenic pathways and clinical presentation (Vannuccini and Petraglia, 2019). It was agreed to define adenomyosis as the presence of ectopic endometrial tissue (endometrial stroma and glands) within the myometrium (Chapron et al., 2020), but not consider it a form of endometriosis

### Anatomical spaces and other locations where endometriosis can be detected

To support the correct application of any future anatomical descriptive system, the locations where endometriosis lesions can be found were defined (Table II). The main resources for these definitions include the ICD (ICD-11, 2020), and publications or textbooks on anatomy (Puntambekar and Manchanda, 2018; Rogers, 2007).

### Endometriosis treatments and interventions

Specific terminology for interventions to treat endometriosis is often used, but not consistently. For endometrioma interventions, definitions were deduced from a recent good practice paper for endometrioma surgery (Working Group of ESGE, ESHRE and WES, 2017 a, Working Group of ESGE, ESHRE and WES, 2017b). Interventions for deep endometriosis were previously defined (Vanhie et al., 2016) and good practice recommendations formulated (Working Group of ESGE, ESHRE and WES Part 2, 2020a; Working Group of ESGE, ESHRE and WES Part 2, 2020b). An overview of the different interventions and their definitions is available in Table III.

The definition of reproductive surgery from the International Glossary on Infertility and Fertility Care was specified towards female patients, as the original definition includes both male and female reproductive surgery (Zegers-Hochschild et al., 2017).

Bowel shaving was previously defined as superficial excision of bowel serosal and subserosal endometriosis (mechanically, with electrosurgery, laser or other energy source) that does not require suturing/closure (Vanhie et al., 2016), but other definitions and interpretations have also been proposed and applied. The working group considered shaving to be a form of partial thickness discoid excision, but agreement on a more specific definition could not be reached. The working group therefore recommends using the more accurate and specific terms included in this terminology and abandon the term ‘shaving’.

With regards to ablation, the term is limited to obliteration of theinner surface of the cyst wall in cases of endometriomas and/or endometriotic lesions in cases of peritoneal endometriosis using electro- orultrasound high frequency-modes, laser, or plasma energy. Nonsurgical treatment options, such as sclerotherapy, can be defined as the destruction of the endometrial tissue using, for example, alcohol installation.

For the different surgical techniques and definitions, it can be considered that surgery can be complete or incomplete, i.e. with visually fibrotic and/or endometriotic lesions left in place. This was not considered a relevant addition to the definitions, but should be included in the patient records.

### Outcome parameters

The lack of internationally agreed outcome parameters for endometriosis interventions largely affects the value of individual studies when attempting to draw conclusions for clinical practice (Meuleman et al., 2011). Specifically for outcomes in endometriosis (pain, recurrence, quality of life [QoL]), their definition may affect the conclusions from the studies. A recently published consensus defines a core outcome set that should be implemented when evaluating potential treatments for endometriosis to standardise outcome selection, collection and reporting (Duffy et al., 2020), yet this outcome set does not include a specific definition of all outcomes. Pain, infertility and QoL are included in the terminology as symptoms or outcomes with previously published definitions (Table IV). With regards to evaluating pain outcomes, a patient-based 11-point Numerical Rating Score, in which the preoperative and post-operative symptoms are given by the patient, allows a better evaluation of the post- operative pain situation as well as the evaluation of de novo pain symptoms possibly associated with a specific type of surgery, when compared with the rating of symptom prevalence and severity by others (physicians, nurses) (Vincent et al., 2010). For QoL outcomes, preference should be given to validated QOL questionnaires, such as the Endometriosis Health Profile Questionnaire (EHP-5 and EHP-30). Recurrence has been defined depending on symptom or lesion recurrence, but a time frame was not included. Further terms included are complications (according to the Clavien-Dindo grading), sequelae and residual disease.

## Discussion

The current paper outlines a list of 49 terms and definitions in the field of endometriosis, as a result of a consensus-based approach. The list includes a definition for endometriosis and its subtypes, different locations, interventions, symptoms and outcomes. The aim of this terminology is to provide a standardised language for the description of endometriosis, to be disseminated and applied widely and to be used as the basis for a new descriptive system for endometriosis. Furthermore, the use of the defined terms should lead to harmonisation in endometriosis research and clinical practice. Further research in endometriosis, its diagnosis and pathogenesis may allow further refinement of the definitions provided.

## WHAT DOES THIS MEAN FOR PATIENTS?

Different definitions are used for endometriosis, endometriosis subtypes, treatments and outcomes. This has significant consequences for research and clinical practice. The current paper is prepared by an international group of experts and lists a number of terms used in endometriosis, with a relevant and appropriate definition. Endometriosis is defined as an inflammatory disease process characterized at surgery by the presence of endometrium-like epithelium and/or stroma outside the endometrium and myometrium, usually with an associated inflammatory process. These definitions should result in harmonisation both in endometriosis research and in clinical practice.
